# Single-Tube Loop-Mediated Isothermal Amplification Assay Targeting the *inlA* Gene for Sensitive Detection of *Listeria monocytogenes* in Food

**DOI:** 10.17113/ftb.64.01.26.9231

**Published:** 2026-02-15

**Authors:** Anna Maraz, Melinda Pazmandi, Kristof Ivan, Agnes Belak

**Affiliations:** 1 Department of Food Microbiology, Hygiene and Safety, Institute of Food Science and Technology, Hungarian University of Agriculture and Life Sciences, Somloi ut 14-16, 1118 Budapest, Hungary; 2 Faculty of Information Technology and Bionics, Pazmany Peter Catholic University, Prater utca 50/A, 1083 Budapest, Hungary

**Keywords:** loop-mediated isothermal amplification (LAMP), *Listeria monocytogenes*, detection, *inlA*

## Abstract

**Research background:**

Several loop-mediated isothermal amplification (LAMP) assays with good performance characteristics have been developed for the detection of *Listeria monocytogenes* in food; however, there are only a few cases in which DNA extraction, amplification and sensing have been performed in a single-tube system.

**Experimental approach:**

The efficiency of DNA extraction by lysis buffers was tested using LAMP. New primer sets for LAMP assays were designed using PrimerExplorer V5 software. The sensitivity and specificity of the LAMP inner primers were determined by optimised PCR. The end-point detection involved gel electrophoresis, turbidity and eriochrome black T (EBT) colour reaction. The sensitivity, specificity and limit of detection (LOD) of the developed LAMP assays were then characterised using *L. monocytogenes*, non-*monocytogenes Listeria* and non-*Listeria* bacterial strains.

**Results and conclusions:**

Both the alkaline cell lysis-based sodium hydroxide and Tris-HCl (HotSHOT)+Tween buffer and the Triton X-100 and sodium azide-based TZ buffer generated amplifiable DNA templates under isothermal conditions for LAMP. However, the TZ buffer produced a significantly higher DNA yield than the HotSHOT+Tween buffer. LAMP primers were designed to target the *hlyA* and *inlA* virulence genes of *L. monocytogenes*. The sensitivity and specificity of the LAMP inner primers were 100 % for both genes; however, the PCR reaction targeting the *inlA* gene generated fewer non-specific PCR products than the *hlyA*-targeting PCR. The sensitivity of the InlA LAMP assay was 100 %, while its specificity was 96 %. The LOD was 500 fg per reaction, which corresponds to 157 genome copy numbers. The combination of DNA extraction, LAMP amplification, and colorimetric endpoint detection in a single tube resulted in a LAMP assay suitable for the detection of *L. monocytogenes* in food under laboratory conditions, with potential for further development for *on-site* detection with microfluidic platforms.

**Novelty and scientific contribution:**

To the best of our knowledge, this is the first report of a LAMP assay targeting the *inlA* gene of *L. monocytogenes*. The developed single-tube LAMP assay is well-suited for integration with microfluidic systems.

## INTRODUCTION

In the global context of foodborne illnesses, bacteria have been identified as the predominant contaminants responsible for most outbreaks worldwide (*1*, *2*). *Listeria monocytogenes* is the leading foodborne pathogen isolated from various food products. Ingestion of contaminated foods allows the bacterium to proliferate, leading to sporadic cases of listeriosis (*3*, *4*).

*L. monocytogenes* isolates originating from food and food processing environments belong mostly to lineage II, which comprises serotypes 1/2a, 1/2c and 3a, and less frequently to lineage I, which includes serotypes 1/2b, 3b, 3c and 4b (*5*). Analysis of epidemiological data has determined that the 1/2a, 1/2b and 4b serotypes of *L. monocytogenes* account for the majority of human outbreaks, despite there being 13 serotypes that have the potential to infect humans. These findings suggest that certain *L. monocytogenes* subtypes are better adapted to food-associated environments and human infection (*6*).

Detection of *L. monocytogenes* in food matrices presents several challenges, such as low numbers and uneven distribution of the cells, presence of background microbiota, stress-induced viable but non-culturable (VBNC) state, complexity of food matrices, biofilm formation or differentiation from other *Listeria* species (*7*-*9*).

Conventional (culture-based) methods are the gold standard for microbiological analysis, as they provide reliable detection and quantification of pathogenic bacteria in foods, from the enrichment to the identification under simple laboratory conditions. Pre-enrichment of food samples in non-selective or selective culture media can increase the number of viable but injured bacterial cells that pose a potential threat to human health to a level that can be detected (*10*). Enrichment steps, including selective enrichment and selective plating, may require an additional period of 8–24 hours before detection or enumeration can be completed. These steps are usually followed by biochemical testing, serological confirmation and identification by conventional and alternative methods (*11*, *12*).

Various standard methods are available for the isolation and detection of *L. monocytogenes* from food products, which are different in the enrichment and detection techniques (*3*). The internationally recognised and applied ISO 11290-1:2017 standard (*13*) consists of a two-step enrichment process involving the incubation of samples (25 g) in half Fraser broth for 24-26 h, followed by inoculation at a 1:100 ratio into full Fraser broth to further enrich the bacterial cultures for 24 h. Pre-enriched and enriched samples are streaked onto selective chromogenic agar plates and presumptive colonies are confirmed.

The use of conventional culture-based methods is time-consuming and labour-intensive (*14*, *15*). Consequently, a wide range of alternative methods have been developed for microbiological food analysis, providing solutions to the limitations of culture-based methods. Molecular DNA amplification techniques such as loop-mediated isothermal amplification (LAMP) play a leading role among them with continuous efforts for development (*11*, *15*).

LAMP was developed by Notomi *et al.* (*16*) at Eiken Chemical Co., Ltd., Tokyo, Japan, and is now used in many areas of microbiology (*17*). LAMP has several advantages, such as simplicity, robustness, low equipment and operation costs, and versatility. DNA amplification does not require heat denaturation before primer attachment, and the reaction is carried out at an isothermal temperature (around 65 °C) using special DNA polymerases with strand displacement activity (*17*-*19*). In the LAMP assay, four different primers recognise six different sites on the target DNA, initiating the amplification reaction. Two additional primers bind to four other sites in the template DNA, which ensures a high degree of specificity. As a result of polymerase activity, hairpin loops of different sizes and cauliflower-like structures comprising multiple loops are formed. The target DNA is amplified 10^9^–10^10^-fold in 15–60 min (*18*).

Mori *et al.* (*20*) found that precipitation of magnesium pyrophosphate during DNA synthesis is proportional to the generated amplicons and can be detected visually or monitored using a real-time turbidimeter. Besides turbidity, endpoint detection can also be achieved using the fluorescent dye calcein (*18*), adenosine triphosphate (ATP) bioluminescence, and colour changes (*21*). Colorimetric assays are very promising, especially the use of metal indicators that detect the decrease in Mg^2+^ concentration during the amplification reaction. Goto *et al.* (*22*) developed the first colorimetric detection based on the reaction of hydroxynaphthol blue (HNB) and Mg^2+^. This method is characterised by a change in colour from purple to blue as the concentration of Mg^2+^ decreases. Oh *et al.* (*23*) applied a new colorimetric sensor based on the reaction of the metal indicator Eriochrome Black T (EBT) and Mg^2+^.

A crucial aspect of developing new LAMP assays and designing suitable primer sets is the selection of an appropriate gene that ensures specificity and sensitivity. For pathogenic microbes, virulence genes are primarily considered as target genes for designing LAMP primers. The virulence genes of *L. monocytogenes* are common to almost all *L. monocytogenes* cell lines, but are typically absent from non-pathogenic *Listeria* species, including the genome of *L. innocua* (*24*). Most virulence determinants of *L. monocytogenes* interfere with the cytoplasmic movement of the infected epithelial cells, involving actin-based motility and cell-to-cell spreading. Adhesion proteins like *Listeria* adhesion protein (LAP) participate in the first step of intracellular infection. LAP has a crucial role in epithelium crossing; however, LAP homologues are present in both pathogenic and non-pathogenic *Listeria* species. The invasion process is directed by several virulence proteins. Internalin A (InlA) and Internalin B (InlB), encoded by the *inlA* and *inlB*, respectively, are responsible for the internalization into the enterocytes and pass through the M-cells of Peyer’s patches. After invasion, bacterial cells are confined within phagosomes inside the enterocytes, which are disrupted by the toxin listeriolysin O (LLO) encoded by *hlyA,* in collaboration with two phospholipases PlcA and PlcB, encoded by *plcA* and *plcB*, respectively. As a result, bacteria are released into the cytosol, where they continue to multiply and spread to other cells. Virulence in *Listeria* is efficiently regulated by the PrfA protein, which controls the expression of most virulence genes, such as *inlA, inlB, hlyA, plcA* and *plcB* (*3*, *25*). Raybourne (*26*) found that the virulence genes *hlyA, actA, mpl, iap* and *inlA* are present in almost all wild-type *L. monocytogenes* isolates; however, Bubert *et al.* (*27*) published that *iap* gene encoding the protein p60 is common to all *Listeria*.

Most publications report on *L. monocytogenes*-specific LAMP assays designed for the detection of the *hlyA* gene, as well as the metalloprotease (Mpl) gene (*28*), the *lmo0753* gene coding for a PrfA-like transcription regulator (*29*), and the iron transport protein gene *feoB* (*30*). As more partial and whole genome sequences of *L. monocytogenes* strains are publicly available, it is possible to select highly conserved virulence genes and increase the specificity of LAMP assay by identifying conserved regions of the selected genes.

Inhibitor-free, fully or partially purified DNA templates are used in conventional LAMP, which provides suitable conditions for standardising the reaction. Several commercial kits are available for template preparation, which differ in their methods of DNA extraction and purification from bacterial cells. In the simplest procedures, nucleic acids are precipitated from the cell lysates with a solvent (ethanol or propanol), followed by RNase treatment, precipitation, or column separation and concentration of DNA (*31*).

The use of commercial DNA isolation kits for DNA extraction from different bacteria often faces the problem that DNA extraction from Gram-positive bacteria is generally less efficient than from Gram-negative ones (*31*, *32*). DNA extraction from bacterial cells requires mechanical or enzymatic disruption of the cell wall before using the kits. Standardization of mechanical cell disruption is not an easy task and instruments for molecular biological purposes, especially for parallel processing of multiple small samples, are not readily available. Proteinase K and/or lysozyme are usually used for enzymatic cell wall disintegration. However, reaction conditions are generally difficult to standardise, and cell wall disintegration is greatly influenced by the properties of the cells being treated. Gram-positive bacteria are much more resistant than Gram-negative ones, and older or stressed cells are also less sensitive than young, fast-growing cells. Proteinase K solution must be inactivated by heat treatment (at 90-100 °C) before the amplification reaction, as it can attack DNA polymerases. Lysozyme is better in this respect, as it does not influence DNA polymerases (*31*-*33*).

Cell disintegration methods are based on two principles (Table 1 (*34*-*37*)). One is the alkaline cell lysis, which is carried out using NaOH, while EDTA-Na_2_ is added to inhibit nucleases. Truett *et al.* (*34*) combined alkaline cell lysis with heat treatment, then adjusted the pH with a neutralizing solution to a value optimal for the PCR reaction (HotSHOT), which resulted in PCR-quality mouse cell extracts. Brewster and Paoli (*35*) further developed the HotSHOT method for extracting DNA from pathogenic bacteria; however, a 2- or 5-fold increase in NaOH and the addition of Tween 20 were required to achieve effective cell lysis in both Gram-negative and Gram-positive bacteria. An advantage of this method compared to the above-mentioned one (*34*) is that a heat treatment of 65 °C is used during cell lysis instead of 95 °C.

**Table 1:** Lysis buffers and treatment conditions used for the preparation of DNA extracts for PCR

Lysis buffer		Active ingredient	Composition	Complementary treatment	Reference
HotSHOT	NaOH		25 mM NaOH, 0.2 mM EDTA-Na_2_	Lysis: 95 °C, 10 min Neutralization: 40 mM Tris-HCl	Truett *et al.* (*34*)
HotSHOT 2× + Tween	NaOH Tween 20		50 mM NaOH, 0.4 mM EDTA-Na_2_, 0.1 % Tween 20	Lysis: 65 °C, 15 min Neutralization: 50 mM HCl, 10 mM Tris-HCl	Brewster and Paoli (*35*)
HotSHOT 5× + Tween	NaOH Tween 20		125 mM NaOH, 1 mM EDTA-Na_2_, 0.1 % Tween 20	Lysis: 65 °C, 15 min Neutralization: 50 mM HCl, 10 mM Tris-HCl	Brewster and Paoli (*35*)
Triton X-100	Triton X-100		2 % Triton X-100	100 °C, 10 min	Agersborg *et al.* (*36*)
TZ	Triton X-100 Na-azide		2 % Triton X-100, 25 mM Na-azide, 0.1 M Tris-HCl, pH=8	Lysis: 100 °C, 15 min	Abolmaaty *et al.* (*37*)

In the second type of cell disintegration technique, the addition of nonionic surfactant Triton X-100 improves the efficiency of cell lysis. Agersborg *et al.* (*36*) established conditions for the lysis of *L. monocytogenes* cells with a solution containing Triton X-100 in combination with a 10-minute heat treatment at 100 °C. Abolmaaty *et al.* (*37*) further developed this method by adding sodium azide and using the solution they called TZ lysis buffer. Brewster and Paoli (*35*) confirmed that cell disintegration with TZ buffer is suitable for the preparation of cell lysates for direct PCR amplification from several bacteria.

Future trends in the development of molecular techniques are to simplify instrumentation, increase the reliability of endpoint detection, and enable *on-site* application (*11*, *38*). Microfluidic biosensors, also known as lab-on-a-chip (LOC), meet these expectations. LOCs can perform a complex laboratory diagnosis on a portable chip, embedding the entire analytical system from the sample preparation to the sensing stage in a single device. The low manufacturing cost allows for mass production and disposability of the devices. The advantage of LOC systems is not only the price, but also their portability, speed, accuracy and automation (*39*).

Microfluidic platforms combine molecular diagnostic techniques with simple, disposable or reusable analytical devices for pathogen detection in food, which meet the affordable, sensitive, specific, user-friendly, rapid and robust, equipment-free, deliverable to end users (ASSURED) criteria, set by the World Health Organisation (WHO) as a diagnostic device standard especially for developing countries (*38*-*40*).

It is advantageous if the DNA template from the enriched food sample is produced in a single tube under closed conditions, especially if a microfluidic chip is used for the detection of foodborne pathogenic bacteria. Another advantage is that there is no need for high heat treatment, *e.g.* 90–100 °C, during or after cell disintegration, as this allows production of DNA extract directly in a heat-sensitive chip. Furthermore, if DNA extraction and amplification take place in a single tube under isothermal conditions, this technique can be directly implemented in microfluidic devices. Closed tube system can decrease the risk of carryover contamination, which is one of the major drawbacks of LAMP (*17*).

Our goal is to implement a single-tube system for cell lysis and amplification, which is suitable for the detection of *L. monocytogenes* by LAMP under laboratory conditions and can be implemented in microfluidic devices. Since LAMP is equally or even slightly more tolerant to inhibitors than PCR (*41*), we focused on the cell lysis and DNA extraction procedures, which were tested and evaluated in PCR. This was followed by designing LAMP assays for the detection of *L. monocytogenes* virulence genes and selecting the best-performing LAMP assay for detection purposes.

## MATERIALS AND METHODS

### Microorganisms used in this study

The list of strains and their origin is shown in Table 2.

**Table 2:** Microorganisms used in this study

No.	Strain	Culture collection/origin
*Listeria monocytogenes* strains		
1	*Listeria monocytogenes* NCAIM B01966	NCAIM
2	*Listeria monocytogenes* CCM 4699 (ATCC 19117)	CCM
3	*Listeria monocytogenes* NCTC 5105 (serovar 3a)	NCTC
4	*Listeria monocytogenes* DMB E ST/10.12. 2	DFMHS/cheese
5	*Listeria monocytogenes* DMB 46	DFMHS
6	*Listeria monocytogenes* DMB H1	DFMHS/meat processing
7	*Listeria monocytogenes* DMB 80	DFMHS/milk
8	*Listeria monocytogenes* DMB H2	DFMHS/meat processing
9	*Listeria monocytogenes* DMB H6	DFMHS/meat processing
10	*Listeria monocytogenes* DMB E 12/10.12. 3	DFMHS
11	*Listeria monocytogenes* DMB 43	DFMHS
12	*Listeria monocytogenes* DMB 8	DFMHS
Non-*monocytogenes Listeria* strains		
1	*Listeria innocua* CCM 4030^T^	CCM
2	*Listeria innocua* DMB 291	DFMHS
3	*Listeria innocua* DMB 217	DFMHS
4	*Listeria innocua* DMB 4191	DFMHS/meat
5	*Listeria innocua* DMB 1969	DFMHS
6	*Listeria innocua* NCAIM B01830	NCAIM
7	*Listeria ivanovii* ssp. *ivanovii* CCM 5884^T^	CCM
8	*Listeria ivanovii* DMB 1149	DFMHS
9	*Listeria ivanovii* DMB 1150	DFMHS
10	*Listeria ivanovii* DMB T7	DFMHS
11	*Listera grayi* DMB 1160	DFMHS
12	*Listera grayi* DMB 1161	DFMHS
13	*Listeria seeligeri* DMB 1153	DFMHS
14	*Listeria welshimeri* CCM 3971^T^	CCM
15	*Listeria welshimeri* DMB 1157	DFMHS
Non-*Listeria* bacterial strains		
1	*Lactococcus lactis* A1	DFMHS
2	*Lactococcus cremoris* B1	DFMHS
3	*Bacillus cereus* PA1	DFMHS
4	*Staphylococcus epidermidis* PA2	DFMHS
5	*Lactobacillus delbrueckii* ssp. *bulgaricus* B397	IDM
6	*Lactobacillus acidophilus* N2	IDM
7	*Pseudomonas fluorescens* CCM 2115^T^	CCM
8	*Pseudomonas lundensis* CCM 3503^T^	CCM
9	*Campylobacter jejuni* ssp. *jejuni* CCM 6214^T^	CCM
10	*E. coli* ATCC 8739	ATCC

CCM=Czech Collection of Microorganisms, Masaryk University, Brno, Czech Republic; ATCC=American Type Culture Collection, Rockville, MD, USA; NCAIM=National Collection of Agricultural and Industrial Microorganisms, Budapest, Hungary; DFMHS=Department of Food Microbiology, Hygiene and Safety, MATE, Budapest, Hungary; NCTC=National Collection of Type Cultures, UK Health Security Agency, Salisbury, UK; IDM=Institute of Dairy Microbiology, Agricultural Faculty, University of Perugia, Perugia, Italy

### DNA isolation from pure bacterial cultures

Tryptic soy broth (TSB; Sigma-Aldrich, Merck, St. Louis, MO, USA) was used as the culture medium for overnight propagation of *Listeria, Bacillus, Staphylococcus, Pseudomonas* and *E. coli*, while lactic acid bacteria were cultivated for 48 h in MRS broth (Biolab Ltd., Budapest, Hungary) under anaerobic conditions (Anaerocult A; Merck KGaA, Darmstadt, Germany) at 30 °C. An overnight propagation of *Campylobacter jejuni* was performed using a Columbia blood agar plate containing 5 % sterile defibrinated sheep blood (Sigma-Aldrich, Merck) at 37 °C in a microaerophilic environment (Anaerocult C; Merck KGaA).

DNA was isolated using MasterPure Complete DNA and RNA Purification Kit (Epicentre, Madison, WI, USA) according to the manufacturer’s instructions.

### Treatment of L. monocytogenes cells with lysis buffers

Overnight culture of *L. monocytogenes* CCM 4699 in TSB (Sigma-Aldrich, Merck) was inoculated into TSB or Fraser enrichment broth (Noack and Co. GmbH, Vienna, Austria) at 10^4^ cell/mL and incubated at 30 °C for 24 h by shaking (190 rpm). A volume of 1 mL culture containing 10^8^ cells was measured into an Eppendorf tube, cells were sedimented by centrifugation at 13 500×*g* for 4 min at 5 °C (Z216-MK microcentrifuge; HERMLE Benchmark, Sayreville, NJ, USA) and the supernatant was discarded. Cells were resuspended in 100 µL lysis buffers (HotSHOT+Tween and TZ) and treated as described in Table 1.

### DNA isolation from cell lysates

Cell debris was sedimented by centrifugation of 100 µL lysed cells (13 500×*g*, 4 min, 5 °C; Z216-MK microcentrifuge; HERMLE Benchmark) and the supernatant was transferred to a new Eppendorf tube. Nucleic acids (NA) were precipitated with equal volume of iso-propanol and sedimented by centrifugation (13 500×*g*, 4 min, 5 °C). The pellet was washed with 70 % ethanol and dried in SpeedVac Vacuum Concentrator (Thermo Fisher Scientific, Seattle, WA, USA). NA were dissolved in 50 µL TE (Tris-EDTA) buffer, 15 µL RNase solution (10 mg/mL) was added, and the mixture was incubated at 37 °C for 30 min. DNA precipitation was repeated, and the purified DNA was dissolved in 50 µL TE buffer. DNA concentration was measured with NanoDrop microvolume spectrophotometer (Thermo Fisher Scientific).

### Calculation of DNA copy number

Cell number of the *L. monocytogenes* suspensions was determined by plating the cells in tryptic soy agar (TSA; Sigma-Aldrich, Merck). Genome copy number of the extracted DNA was calculated by the Staroscik Copy number calculator of Technology Networks (*42*):


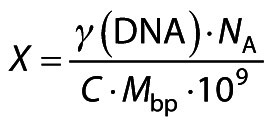
 /1/

where *X* is the number of copies per µL, *γ*(DNA) is the DNA concentration (ng/µL), *N*_A_ is Avogadro’s number (6.0221·10^23^ mol^-1^), *C* is the genome size of the organism expressed in number of base pairs (bp), and *M*_bp_ is the average mass of a base pair (650 Da).

According to the NCBI database, genome size of *L. monocytogenes* CCM 4699 (ATCC 19117) strain is 2 951 805 bp (Genome assembly ASM30702v1 (*43*)).

### LAMP assay targeting the hlyA gene

The total reaction volume was 25 µL containing the components as described by Tang *et al.* (*44*). The LAMP reaction consisted of a 60-minute amplification step at 65 °C and a 10-minute enzyme inactivation step at 80 °C.

### Detection of the amplicons by gel electrophoresis

LAMP products were separated in 1 % agarose gel (SeaKem LE Agarose; Lonza, Basel, Switzerland) by electrophoresis at 120 V for 45 min. DNA bands were visualised by ethidium bromide staining.

### Development of LAMP assays

PrimerExplorer V5 software (*45*, *46*) was used to design LAMP primer sets specific to the genes encoding the listeriolysin O (*hlyA*) and internalin A (*inlA*) of *L. monocytogenes*. The nucleotide sequences of the *hlyA* and *inlA* of *L. monocytogenes* strains N53-1 (accession number: HE999705; GenBank, NCBI) and NRRL B-33220 (accession number: DQ844405, GenBank, NCBI), respectively (*43*), provided the basis for the primer design.

For the detection of these genes, LAMP primer sets were designed, and the sensitivity and specificity of the LAMP inner primers (FIP and BIP) with an optimised PCR reaction were calculated. For the PCR detection of these genes, inner PCR primers (F2-B2) were designed based on the nucleotide sequences of the inner LAMP primers FIP and BIP.

#### HlyA-old primer sets

Primers (5’-3’) for the LAMP reaction were (*44*): F3-H-old: TTGCGCAACAAACTGAAGC; B3-H-old: GCTTTTACGAGAGCACCTGG; LF-H-old: TAGGACTTGCAGGCGGAGATG; LB-H-old: GCCAAGAAAAGGTTACAAAGATGG; FIP: CGTGTTCTTTTCGATTGGCGTCTTTTTTTCATCCATGGCACCACC; and BIP: CCACGGAGATGCAGTGACAAATGTTTTGGATTTCTTCTTTTTCTCCACAAC.

Inner primers (5’-3’) for the PCR reaction were: F2-H-old: TTTCATCCATGGCACCACC; and B2-H-old: GGATTTCTTCTTTTTCTCCACAAC.

#### HlyA-new primer sets

Primers (5’-3’) for the LAMP reaction were: F3-H-new: GTCTCAGGTGATGTAGAACT; B3-H-new: TGTCTTTTAGGAAGTTTGTTGT; LF-H-new: TTGCGGAACCTCCGTAAATTAC; LB-H-new: AAGGCGCTACTTTTAATCGAGAAAC; FIP: CCGTCGATGATTTGAACTTCATCTTTCAAAAATTCTTCCTTCAAAGCC; and BIP: CAACCTCGGAGACTTACGAGAATAAGCAATGGGAACTCCT.

Inner primers (5’-3’) for the PCR reaction were: F2-H-new: TCAAAAATTCTTCCTTCAAAGCC; and B2-H-new: ATAAGCAATGGGAACTCCT.

#### InlA primer sets

Primers (5’-3’) for the LAMP reaction were: F3-I: TGCCAGCAAATGATATTACG; B3-I: TGCTTTTGAATTATAAGGGTCAT; LF-I: GGTGGTGCCACAGGATTTT; LB-I: GAAGCAACACATCTAACACATCAAC; FIP: CTCCGTTATTTGTAGTCGGCGGCTGTACGCTCAATTCACGA; and BIP: CCACCTTCCGCAAATATACCTGGTTCATTGTACTTGTTGTGCT.

Inner primers (5’-3’) for the PCR reaction were: F2-I: CTGTACGCTCAATTCACGA; and B2-I: GTTCATTGTACTTGTTGTGCT.

#### PCR reaction

Composition of the PCR mixture (*V*=25 μL) was: 0.2 μM of each F2 and B2 primer pair, 0.1 mM dNTP, 1×*Taq* polymerase buffer, 0.7 mM MgCl_2_, 0.024 U/µL *Taq* DNA polymerase (New England Biolabs, Ipswich, MA, USA) and 2 ng/µL template DNA. Parameters of the PCR reaction were: (*i*) pre-denaturation at 95 °C for 5 min, (*ii*) denaturation at 95 °C for 20 s, (*iii*) primer annealing at 52 °C for 30 s, (*iv*) elongation at 72 °C for 30 s (steps (*ii*), (*iii*) and (*iv*) were repeated 35 times), and (*v*) final elongation at 72 °C for 3 min.

#### InlA LAMP assay with EBT endpoint detection

Composition of the LAMP mixture (*V*=25 μL) was: F3: 0.2 µM, B3: 0.2 µM, FIP: 1.6 μM, BIP: 1.6 µM, LF: 0.8 µM, LB: 0.8 µM, dNTP: 1.2 mM, betaine: 0.6 M, Thermopol reaction buffer (New England Biolabs): 1×, MgSO_4_: 6 mM, *Bst* 2.0 Warm Start DNA polymerase (New England Biolabs): 0.32 U/µL, Eriochrome® black T (EBT): 120 µM, DNA extract: 2 ng/µL of reaction or cell lysate: 0.04 µL/µL of reaction.

The LAMP reaction consisted of a 60-minute amplification step at 65 °C and a 10-minute enzyme inactivation step at 80 °C.

### Determination of performance characteristics of LAMP assays

#### Determination of sensitivity and specificity

DNA was isolated from ten *L. monocytogenes*, ten non-*monocytogenes Listeria* and ten non-*Listeria* bacterial strains and then the purified genomic DNA was amplified with the LAMP assay applying the EBT endpoint detection.

Sensitivity (SE/%) and specificity (SP/%) were calculated using the following equations:


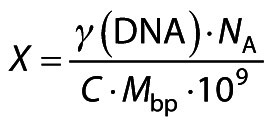
 /2/


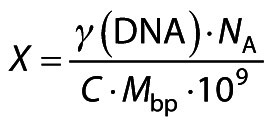
 /3/

where *N*_+_ is the number of true positives, *N*_−_ is the number of true negatives, *P*_A_ is the number of positives obtained by LAMP (alternative) method, and *N*_A_ is the number of negatives obtained by LAMP (alternative) method.

#### Determination of limit of detection

The limit of detection (LOD) was determined in two ways: (*i*) by determining the lowest detectable amount of DNA (copy number): decimal dilution series was prepared from the purified template DNA and the lowest DNA concentration that gave a positive result in the LAMP assay was determined. Considering the genome size of the *L. monocytogenes* CCM 4699 strain, the copy number was calculated as described above, and (*ii*) determining the lowest detectable viable cell number: cell suspensions were prepared from 24-hour cultures of *L. monocytogenes* grown in TSB or Fraser enrichment broth (30 °C, 190 rpm), which were treated with TZ buffer for 15 min at 65 and 100 °C both before and after decimal dilution. The lowest cell number that gave a positive result in the LAMP assay was determined. Experiments were performed in triplicate and in two independent replications.

### Influence of E. coli on the LOD of InlA LAMP

*E. coli* NCAIM B01909 was cultivated in TSB at 30 °C for 24 h with shaking (190 rpm), and a suspension of 10^8^ cell/mL was prepared, which was added at a 1:10 ratio to the TSB and Fraser broth cultures of *L. monocytogenes* CCM 4699 (10^8^ cell/mL). Decimal dilution series were prepared, lysed by TZ buffer at 65 and 100 °C, respectively, and the LOD was determined.

## RESULTS AND DISCUSSION

### Development of a single-tube system for cell lysis, DNA extraction and end-point detection in L. monocytogenes

**Fig. 1:**
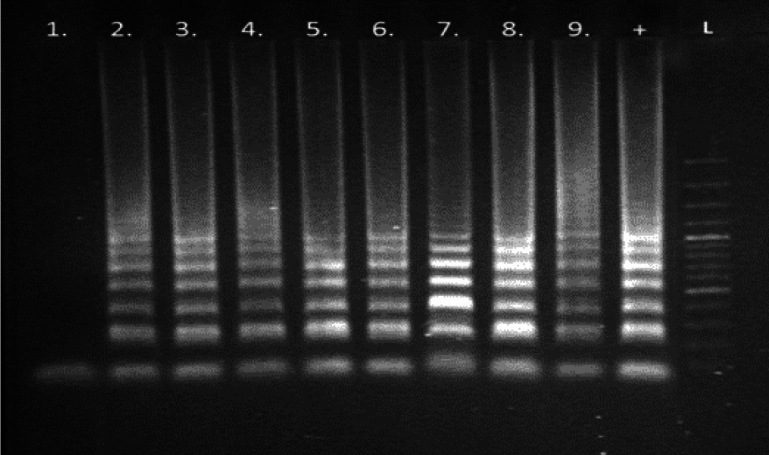
Effect of lysis buffers on the HlyA-old LAMP (loop-mediated isothermal amplification) reaction in original (1×) and diluted (1/10, 1/30) concentrations as detected by gel electrophoresis. Target gene: *hlyA* of *L. monocytogenes* CCM 4699. Template DNA was isolated with the use of MasterPure Complete DNA and RNA Purification Kit. Lanes 1–3=HotSHOT 5×+Tween (1×), (1/10), (1/30); lanes 4–6=HotSHOT 2×+Tween (1×), (1/10), (1/30); lanes 7–9=TZ (1×), (1/10), (1/30). +=positive control (template DNA in TE (Tris-EDTA) buffer), L=100 bp dsDNA ladder. Composition of HotSHOT and TZ buffers is shown in Table 1.

The effect of the alkaline lysis buffer HotSHOT+Tween and the Triton X-100 and sodium azide-based TZ buffer on the LAMP reaction were determined at their original concentrations (*35*, *37*) as well as at 3× and 10× dilutions. A volume of 1 µL (50 ng) of purified template DNA, extracted from *L. monocytogenes* CCM 4699, was added to 10 µL of lysis buffers (Table 1) and used as a template in the LAMP mixtures targeting the *hlyA* gene (*44*). Separation of the reaction products by gel electrophoresis is shown in Fig. 1, which indicates that the HotSHOT 5×+Tween lysis buffer inhibited the LAMP reaction at the original concentration, but not at the diluted concentrations. In contrast, the HotSHOT 2×+Tween and TZ buffers had no inhibitory effect at all. The same results were obtained using turbidity for endpoint detection. We can conclude that using up to 10 µL of the template in HotSHOT 2×+Tween and TZ lysis buffers per LAMP reaction does not inhibit the amplification.

Next, we investigated whether lysing *L. monocytogenes* CCM 4699 cells with HotSHOT+Tween and TZ lysis buffers provides amplifiable DNA templates for the LAMP reaction. For the amplificability test, 1 µL of DNA solution (50 ng) extracted from the cell lysates or 1 µL of cell lysates was used as templates for the LAMP reaction. As shown in Table 3, positive results were obtained for every cell lysate and DNA extract using turbidity or gel electrophoresis for endpoint detection. Treating the cells with lysozyme prior to buffer administration did not significantly affect the results. We investigated whether reducing the temperature of the cell lysis in TZ buffer from 100 to 65 °C would result in adequate cell disintegration and DNA extraction. The results in Table 3 show that heat treatment at 65 °C for 15 min is sufficient to yield amplifiable template DNA, which is advantageous for microfluidic application.

**Table 3:** Amplificability of DNA extracted from cell lysates and that of the cell lysates of *L. monocytogenes* CCM 4699 obtained with HotSHOT+Tween and TZ lysis buffers

Lysis buffer	Treatment	Template	LAMP reaction	
			Turbidity	Gel electrophoresis
HotSHOT 2×+Tween	-	DNA	+	++
HotSHOT 5×+Tween	-	DNA	+	++
TZ (100 °C, 15 min)	-	DNA	+	++
TZ (65 °C, 15 min)	-	DNA	+	++
HotSHOT 2×+Tween	L	DNA	+	+
HotSHOT 5×+Tween	L	DNA	+	+
TZ (100 °C, 15 min)	L	DNA	+	++
TZ (65 °C, 15 min)	L	DNA	+	++
HotSHOT 2×+Tween	-	Cell lysate	+	++
HotSHOT 5×+Tween	-	Cell lysate	+	++
TZ (100 °C, 15 min)	-	Cell lysate	+	+++
TZ (65 °C, 15 min)	-	Cell lysate	+	++
HotSHOT 2×+Tween	L	Cell lysate	+	+++
HotSHOT 5×+Tween	L	Cell lysate	+	++
TZ (100 °C, 15 min)	L	Cell lysate	+	+++
TZ (65 °C, 15 min)	L	Cell lysate	+	++
Positive control	-	Purified DNA	+	++

Target gene: *hlyA* of *L. monocytogenes*. L=lysozyme treatment (6 mg/mL, 37 °C, 30 min) before adding the buffers; +=weak turbidity/amplicon patterns, ++=turbidity/amplicon patterns with medium intensity, +++=strong turbidity/amplicon patterns. Composition of HotSHOT and TZ buffers is shown in Table 1

The efficiency of DNA extraction using the HotSHOT+Tween and TZ lysis buffers was determined by measuring the amount of DNA extracted from the cell lysates. The genome copy number in the extracted DNA was calculated based on the known genome size of *L. monocytogenes* CCM 4699. The ratio of the genome copy number per mL of the extracted DNA to the cell number per mL of the initial cell suspension indicated the efficiency of the DNA extraction. The best result was obtained with the TZ lysis buffer, extracting up to 15 % of the genomic DNA. Much smaller amounts of DNA were extracted from the cells using the HotSHOT+Tween buffer, with only around 5 % of the genomic DNA present in the cell lysates. It should be mentioned that the efficiency of DNA extraction in the cell lysates exceeds the calculated values, as significant losses occur during DNA purification and concentration, particularly in small volumes (*V*≤100 µL).

The main conclusion is that all three lysis buffers result in amplifiable DNA template, with TZ lysis buffer being the most efficient in terms of the amount of extracted DNA. The amplification and sensing reactions of the LAMP assay can be performed in the same tube as the cell lysis when using HotSHOT 2×+Tween and TZ lysis buffers.

### Designing LAMP primer sets for the detection of L. monocytogenes virulence genes

For the detection *of L. monocytogenes* in food, environmental and human samples, conventional and multiplex PCR are the most frequently used molecular techniques, typically targeting virulence genes (*3*). Most LAMP assays are also based on the detection of *L. monocytogenes* virulence genes. However, LAMP requires a much more extensive primer design than PCR, as it uses four or six different primers for amplification, which target six different regions of the gene.

In our previous work, we used a LAMP assay for the detection of *L. monocytogenes* strains based on the amplification of *hlyA* gene (*44*). However, in some cases we obtained false positive or negative results, even when the respective negative or positive controls worked properly. Consequently, in the development of a novel LAMP assay, we targeted different regions of the *hlyA* gene using LAMP primers and also included the *inlA* virulence gene. To detect these genes, we designed LAMP primer sets and determined the sensitivity and specificity of the LAMP inner primers, FIP and BIP, with an optimised PCR reaction. For this reaction, we designed inner PCR primer pairs F2-B2, based on the nucleotide sequences of the inner LAMP primers. Sensitivity and specificity of the PCR primers were tested against 12 *L. monocytogenes* and 15 other *Listeria* strains, respectively, which were reliably identified by phenotypic and molecular methods (Table 2). Results summarised in Table 4 indicate that the HlyA-new and InlA PCR primer pairs generated specific PCR products (in the range of 100–200 bp) for all *L. monocytogenes* strains, while the PCR reaction was negative for one strain with the HlyA-old primer pair. The HlyA-new and InlA PCR reactions did not yield amplicons of the expected size for any of the 15 non-*monocytogenes Listeria* strains; however, non-specific PCR products could be visualised by gel electrophoresis in all three PCR reactions in rare cases. PCR products of various sizes and low intensity appeared primarily in *L. innocua* and *L. ivanovii* strains, although their visibility was different in the three replicates in several cases. Based on the results, the calculated sensitivity and specificity of both the HlyA-new and InlA PCR reactions were 100 %, while these were only 92 and 87 % for the HlyA-old, respectively. Considering that the InlA PCR reaction generated the fewest non-specific products, we selected the InlA LAMP assay for further experiments.

**Table 4:** Results of PCR using F2-B2 internal PCR primers targeting the *hlyA* and *inlA* genes of *L. monocytogenes* strains

			PCR	
No.	Strain	HlyA-old	HlyA-new	InlA
	*L. monocytogenes* strains			
1	*L. monocytogenes* CCM 4699	+	+	+
2	*L. monocytogenes* NCAIM B01966	+	+	+
3	*L. monocytogenes* NCTC 5105	+	+	+
4	*L. monocytogenes* DMB E ST/10.12. 2	+	+	+
5	*L. monocytogenes* DMB E 12/10.12. 3	+	+	+
6	*L. monocytogenes* DMB H1	+	+	+
7	*L. monocytogenes* DMB H2	+	+	+
8	*L. monocytogenes* DMB 80	+	+	+
9	*L. monocytogenes* DMB H6	+	+	+
10	*L. monocytogenes* DMB 43	+	+	+
11	*L. monocytogenes* DMB 46	+	+	+
12	*L. monocytogenes* DMB 8	−	+	+
	Non-*monocytogenes Listeria* strains			
1	*L. innocua* NCAIM B01830	−	−	−
2	*L. innocua* DMB 1969	−	(+)	−
3	*L. innocua* CCM 4030^T^	−	(+)	−
4	*L. innocua* DMB 4191	−	−	−
5	*L. innocua* DMB 291	−	−	−
6	*L. innocua* DMB 217	−	−	−
7	*L. grayi* DMB 1161	−	−	−
8	*L. grayi* DMB 1161	−	−	−
9	*L. ivanovii *ssp*. ivanovii* CCM 5884^T^	+	−	−
10	*L. ivanovii* DMB 1150	+	−	−
11	*L. ivanovii* DMB T7	(+)	(+)	(+)
12	*L. ivanovii* DMB 1149	(+)	(+)	(+)
13	*L. seeligeri* DMB 1153	−	−	−
14	*L. welshimeri* CCM 3971^T^	−	−	−
15	*L. welshimeri* DMB 1157	−	−	−
NC	Negative control (without template DNA)	−	−	−

Template DNA was isolated with the use of MasterPure Complete DNA and RNA Purification Kit. +=positive PCR reaction, −=negative PCR reaction, (+)=non-specific PCR reaction

It is worth noting that the higher primer annealing temperature in the LAMP than in the PCR reaction (65 *vs* 52 °C) can significantly reduce non-specific primer binding and the generation of false positive LAMP products.

### Application of the eriochrome black T for endpoint detection of LAMP assay

Biosensors for LAMP with turbidity-based real-time monitoring and endpoint detection are very simple and widespread. The drawback of applying these systems in microfluidic devices is that visual assessment is subjective and uncertain, and nephelometric microsensors are not yet commercially available. Colorimetric endpoint detection seems more convenient and cheaper in LAMP, especially in microfluidic sensors.

**Fig. 2:**
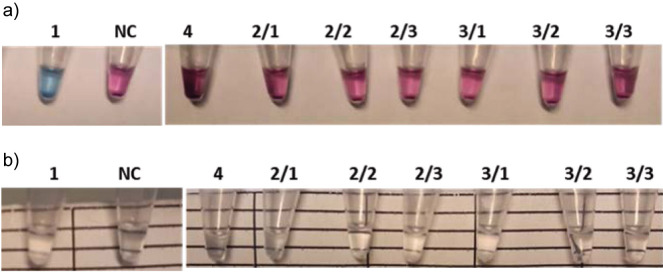
Detection of *L. monocytogenes* (1) and non-*monocytogenes Listeria* (4, 2/1, 2/2, 2/3, 3/1, 3/2, 3/3) strains by InlA LAMP (loop-mediated isothermal amplification) assay using: a) EBT (eriochrome black T) endpoint reaction (blue=positive, purple=negative) and b) turbidity endpoint reaction (turbid=positive, clear=negative). Strains: 1=*L. monocytogenes* NCAIM B01966; 2/1, 2/2, 2/3=*L. innocua* CCM 4030^T^; 3/1, 3/2, 3/3=*Listeria welshimeri* CCM 3971^T^; 4=*L. ivanovii* ssp. *ivanovii* CCM 5884^T^; NC=negative control (LAMP reaction mixture without template DNA). Template DNA was isolated with the use of MasterPure Complete DNA and RNA Purification Kit

We tested the robustness of the hydroxynaphthol blue (HNB) colorimetric detection method (*22*) and found that although the colour change was easily detected, the reaction needed to be optimised and standardised for the conditions of each LAMP reaction. The HNB concentration and LAMP reaction time were particularly important factors (data not shown). We also investigated the applicability of another metal indicator, eriochrome black T (EBT), for colorimetric endpoint detection (*23*) and compared it with the turbidity visualisation, using the InlA LAMP assay, and including *L. monocytogenes* and non-*monocytogenes Listeria* strains in the tests. Results in Fig. 2 show that *L. monocytogenes* strains were positive with both the EBT (Fig. 2a) and turbidity (Fig. 2b) reactions. For non-*monocytogenes Listeria* strains, the EBT reaction was clearly negative, while turbidity was only rarely weakly positive in one of the three parallel samples (2/3 and 3/1). Therefore, we applied EBT endpoint detection in the subsequent LAMP experiments.

### Evaluation of the InlA LAMP assay developed for the detection of Listeria monocytogenes

Performance characteristics of the InlA LAMP assay were evaluated using ten strains of each of *L. monocytogenes*, non-*monocytogenes Listeria* and non-*Listeria* (Table 2). Sensitivity, specificity, and LOD were calculated as the most critical performance characteristics.

**Fig. 3:**
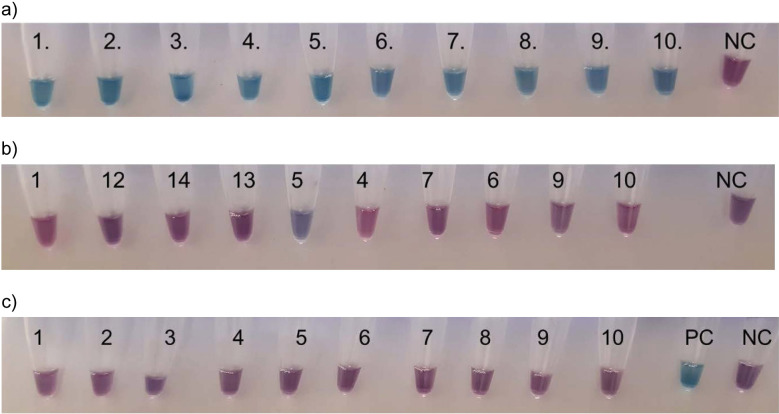
Positive and negative InlA LAMP (loop-mediated isothermal amplification) results testing of: a) ten *L. monocytogenes*, b) ten non-*monocytogenes Listeria* and c) ten non-*Listeria* bacterial strains by EBT (eriochrome black T) endpoint reaction. Template DNA was isolated with the use of MasterPure Complete DNA and RNA Purification Kit. Numbers indicate the strains listed in Table 2. NC=negative control (LAMP reaction mixture without template DNA); PC=positive control (LAMP reaction with *L. monocytogenes* CCM 4699 template DNA)

Fig. 3 shows the results obtained with *L. monocytogenes* (Fig. 3a), non-*monocytogenes Listeria* (Fig. 3b), and non-*Listeria* bacterial strains (Fig. 3c), presenting typical series of EBT reactions.

For sensitivity (SE), the ratio of the number of *L. monocytogenes* strains resulting positive in the LAMP test to the number of true positives was determined, while in the case of specificity (SP), the ratio of the number of non-*monocytogenes Listeria* and non-*Listeria* bacterial strains that gave negative LAMP results to the true negatives was calculated.

The sensitivity of the InlA LAMP assay was 100 %, as all 10 *L. monocytogenes* strains gave positive LAMP results in repeated experiments. This finding is consistent with the results obtained in the InlA PCR reaction. The specificity was 92 % for the non-*monocytogenes Listeria* strains, as 8 % of the LAMP tests were false positives; it was 100 % for the non-*Listeria* bacteria, and 96 % when all non-*L. monocytogenes* strains were considered collectively. In some cases, one of the three parallel reactions was positive, likely due to a slight decrease in Mg^2+^ concentration in the reaction mixture. This is because non-specific amplification can occur in the presence of a substantial amount of DNA released from the background microbiota (*47*, *48*). As shown by Francois *et al.* (*19*), weak amplification of template DNA can also occur in the absence of the target gene. Furthermore, the negative control sample can be significantly amplified if sample preparation conditions (temperature and time) and the amplification period are not optimal. Notably, the false positive results did not originate from the same *Listeria* species that produced weak, non-specific PCR products in the InlA PCR reaction.

LOD refers to the smallest amount of template DNA and the corresponding calculated genome copy number, as well as the smallest amount of living cell count that can be detected by the applied LAMP assay (*19*). The lowest detectable amount of DNA by LAMP (LOD) was determined using decimal dilutions of DNA extracted from *L. monocytogenes* CCM 4699, with the last positive result at the 10^-5^ dilution. Based on this result, the calculated amount of genomic DNA that can be reproducibly detected in the LAMP reaction (*V*=25 µL) is 500 fg, which corresponds to 157 genome copy numbers. The calculated LOD per unit reaction volume is 20 fg/µL, or 6.3 copy/µL. This is close to the LOD value of LAMP obtained by Nathaniel *et al.* (*29*) for the *lmo0753* gene of *L. monocytogenes* and by Francois *et al.* (*19*) for *Salmonella*; however, it is ten times lower than that obtained by Busch *et al.* (*28*) for LAMP developed for the *mpl* gene of *L. monocytogenes*. Oh *et al.* (*23*) reported a similar LOD using LAMP with EBT sensing for the detection of *E. coli* O157:H7 in a centrifugal microfluidic device.

### Detection of L. monocytogenes with InlA LAMP by direct amplification from cell lysate

Traditional detection of *L. monocytogenes* in food, environmental and human samples by LAMP includes culturing the samples in a selective medium, followed by DNA isolation from the typical colonies for detection. When the number of *L. monocytogenes* cells is low, a one- or two-step enrichment process is initiated using appropriate culture media, applying a quantity of food samples corresponding to the limit value. These samples are then cultured to assess the presence or absence of *L. monocytogenes*. This can be achieved by further cultivation in a selective culture medium and confirmation of the typical colonies or identification by culture-based or culture-independent (*e.g.* genomic) techniques (*11*, *12*, *15*).

It is necessary to check whether enrichment media inhibit the reaction at the applied concentrations. Since Fraser selective broth is one of the most frequently used and well-established media for selective enrichment of *L. monocytogenes* in food (*13*), its inhibitory effect on LAMP was investigated. According to the results, adding the maximum template volume (*V*=4 µL) to the reaction mixture did not inhibit the LAMP reaction. This indicates that the enriched culture can be used directly for cell lysis, and then as a template for the LAMP reaction. Since enrichment occurs in two consecutive steps, the inhibitory effect of the food matrix on the LAMP reaction should not be expected, as the first broth (half-Fraser or other) containing the food sample is diluted 100-fold in the second (Fraser) broth.

Since the TZ buffer proved to be the most efficient of the tested lysis buffers, it was selected for the disintegration of *L. monocytogenes* cells. The applicability of the resulting cell lysates for direct detection of *L. monocytogenes* using the InlA LAMP assay was then tested. Cultures of *L. monocytogenes* CCM 4699 grown in either TSB or Fraser enrichment medium were lysed in TZ buffer. The efficiency of cell lysis at 100 and 65 °C for 15 min was compared by the determination of LOD values. Results are summarised in Table 5.

**Table 5:** Limit of detection (LOD) values of the InlA LAMP (loop-mediated isothermal amplification) assay determined by *L. monocytogenes* CCM 4699 single cultures or co-cultures with *E. coli* ATCC 8739, prepared in tryptic soy broth (TSB) and Fraser enrichment broth

Temperature/°C	Culture medium	Species	Lysis with buffer	*N*/(cell/mL)	*N*/(cell/reaction) *V*=25 µL	*N*/(cell/µL)
	TSB	*L. monocytogenes*	Before dilution	10^4^	100	4
	TSB	*L. monocytogenes*	After dilution	10^3^	10	0.4
100	TSB	*L. monocytogenes+E. coli*	After dilution	10^4^	100	4
	Fraser	*L. monocytogenes*	Before dilution	10^5^	1000	40
	Fraser	*L. monocytogenes*	After dilution	10^4^	100	4
	Fraser	*L. monocytogenes+E. coli*	After dilution	10^4^	100	4
	TSB	*L. monocytogenes*	Before dilution	10^5^	1000	40
	TSB	*L. monocytogenes*	After dilution	10^4^	100	4
65	TSB	*L. monocytogenes+E. coli*	After dilution	10^5^	1000	40
	Fraser	*L. monocytogenes*	Before dilution	10^6^	10000	400
	Fraser	*L. monocytogenes*	After dilution	10^4^	100	4
	Fraser	*L. monocytogenes+E. coli*	After dilution	10^4^	100	4

The cultures were lysed in TZ buffer (composition is in Table 1) at 100 °C and 65 °C for 15 min

Adding diluted suspensions of TSB cultures lysed by TZ buffer at 100 °C as templates in the InlA LAMP reaction resulted in an LOD similar to that obtained with purified DNA. The LOD was in the range of 100 cells per reaction, indicating that *L. monocytogenes* could be reliably detected in suspensions of ≥10^4^ cell/mL. Cell lysis with TZ buffer at 65 °C increased the LOD by one order of magnitude. In Fraser cultures, the LOD was one order of magnitude higher than in TSB at both lysis temperatures, which could be attributed to the presence of several inhibitory compounds in the Fraser broth that make the cells tolerant to the lysing effect of the TZ buffer.

The number of *L. monocytogenes* cells at the end of the enrichment period depends on the initial number of cells in the sample and their ability to grow in the pre-enrichment (half-Fraser) and enrichment (Fraser) broths (*49*). Therefore, it is expected that *L. monocytogenes* will need to be detected in positive samples with different cell numbers. To test the effect of cell numbers on the LOD, decimal dilution series of TSB and Fraser cultures (10^8^ cell/mL) were prepared in the corresponding culture medium. The dilution series members were then lysed individually with TZ buffer at 65 and 100 °C, and the lysed samples were used as templates for the InlA LAMP assay. Lysing the dilution series members individually resulted in a one-order-of-magnitude decrease in the LOD most of the time for both the TSB and Fraser enrichment media at 65 and 100 °C (Table 5). It can be concluded that the efficiency with which TZ buffer lyses cells increases at concentrations below 10^8^ cell/mL at both 100 and 65 °C, and that *L. monocytogenes* can be reliably detected in samples even at low cell number (≤10^4^ cell/mL) after enrichment.

During the selective enrichment of *L. monocytogenes*, non-*Listeria* cells present in the food sample may also grow at a low rate. Therefore, we tested whether adding *E. coli* cells at a ratio of 1:10 to *L. monocytogenes* TSB and Fraser broth cultures affects the LOD values. Results in Table 5 show that the presence of *E. coli* cells during cell lysis has practically no influence on the LOD values, confirming high specificity and robustness of the developed InlA LAMP assay.

As the continuation of the present work, validation of the developed InlA LAMP assay according to the ISO 16140-2:2016 (*50*) is the next step using different food categories artificially inoculated and naturally contaminated with *L. monocytogenes*.

## CONCLUSIONS

The selection and improvement of inhibitor-free cell lysing processes for DNA extraction and amplification offer a new approach for the development of a single-tube loop-mediated isothermal amplification (LAMP) assay that can be directly applied to detect the most important foodborne pathogen, *Listeria monocytogenes*, in food after enrichment. It has been demonstrated that the selected TZ lysis buffer, when added at a volume equivalent to one-sixth of the LAMP reaction volume, does not inhibit the amplification process and does not affect the eriochrome black T (EBT) sensing reaction.

The use of genome sequencing data analysis and testing the LAMP primer specificity and sensitivity by PCR reaction enabled the selection of *InlA* as the target gene for detection. It was shown that the sensitivity of the hlyA LAMP assay could be improved and that the highest specificity was achieved using the *InlA* target gene. To the best of our knowledge, this gene has not been used previously in a LAMP assay.

The developed single-tube InlA LAMP assay has been shown to be suitable for the detection of enriched *L. monocytogenes* cultures under laboratory conditions. Furthermore, it has the potential to be further developed for *on-site* detection with microfluidic devices.
